# Aneurysmal Bone Cyst Presenting as Fragility Fracture: A Case Report Focused on the Rehabilitation Approach

**DOI:** 10.7759/cureus.21145

**Published:** 2022-01-12

**Authors:** Ana Teixeira-Vaz, Mariana Santiago, Mafalda Oliveira, Ana Isabel Silva

**Affiliations:** 1 Physical Medicine and Rehabilitation Department, Centro Hospitalar Universitário de São João, Porto, PRT

**Keywords:** aneurysmal bone cyst, case report, rehabilitation, pediatric fractures, pediatric rehabilitation

## Abstract

Aneurysmal bone cysts are rare lesions, comprising one to six percent of primary bone tumors. Despite benign, those may be locally aggressive. We report a pediatric case with an atypical presentation. The patient was a seven-year-old boy, admitted to an emergency room due to right inguinal pain, without a history of trauma. The symptoms had acute onset and worsened gradually for five days. Radiographs revealed a cystic lesion on the proximal right femur and two longitudinal fractures. After further diagnostic work-up and given the probable diagnosis of an aneurysmal bone cyst, surgical treatment was performed. The diagnosis was then confirmed by histopathological analysis. After surgery, the patient maintained severe pain, having an important range of motion (ROM) and muscular strength reduction on the affected limb. As so, the patient engaged in a daily tailored Physical Medicine and Rehabilitation (PMR) program, for four months. After concluding the treatment plan, the patient was asymptomatic: recovery of both ROM and muscular strength was achieved, as well as the ability to return to previous daily-life activities. This is a paradigmatic case in which a rare condition with a rare presentation was displayed after several others were ruled out, requiring a multidisciplinary approach.

## Introduction

Aneurysmal bone cysts are benign lesions comprised of blood-filled channels, that may arise *de novo*, secondarily to other benign or malignant bone tumors, or in relation to bone trauma [1,2]. Although multiple theories have been proposed to describe their etiopathogenesis, it remains undetermined [3]. 

Despite having a benign nature, aneurysmal bone cysts can be locally aggressive. Recurrence rates of up to 70% have been reported, generally in children with open growth plates, and within the first two years after surgical removal [1,2]. 

Epidemiologically, aneurysmal bone cysts usually develop during the first two decades of life, with no clear gender preference, most often on the metaphysis of long bones [4-6]. These rare lesions, which comprise one to six percent of primary bone tumors, display distinctive features [2,7].

Clinical symptoms are usually indolent, but possibly rapidly progressive. Pain is the most common symptom, usually insidious, progressing from several weeks to months. Nonetheless, a minority of patients present with a sudden onset of pain (as a result of a pathological fracture). Swelling, stiffness, deformity and decreased range of motion (ROM) can also be reported [5].

Radiographs, computed tomography (CT), and magnetic resonance imaging (MRI) are suitable diagnostic methods; nevertheless, MRI is the most appropriate method to define fluid areas and to rule out other diagnoses. Pathologic features consist of blood-filled spaces, separated by osteoid tissue [1,6]. USP6 rearrangements can be observed in up to 75% of aneurysmal bone cysts [5]. Despite USP6 action as an oncogene, it is believed that the tumor has no malignant potential [5]. Its identification can be useful for excluding morphological mimics, namely telangiectatic osteosarcoma and other malignant lesions complicated by secondary aneurysmal bone cysts [8]. 

Therapeutic options are variable and dependent on the location and size of the lesion, but the standard of care is surgical resection [6]. Several other treatment options are nowadays available, specifically selective arterial embolization [9], sclerotherapy [10], and denosumab [11]; regardless of working successfully in some cases, efficiency studies are still warranted. 

Physical Medicine and Rehabilitation (PMR) programs have a significant role after surgical intervention, targeting pain control, scar desensitization, ROM recovery, muscular strengthening, functional limb reeducation, and balance and gait training [12]. 

We report a pediatric case of an unusual presentation of a large aneurysmal bone cyst, in which a multidisciplinary approach was adopted.

## Case presentation

A 7-year-old boy, without any relevant background and/or a history of trauma, was admitted to the emergency room due to right inguinal pain that extended to all right inferior limb (RIL). The symptoms had acute onset, worsened gradually for five days, and were refractory to anti-inflammatory/analgesic drugs. A giving-away feeling and gait claudication were also reported. Physical examination revealed severe pain on active and passive RIL mobilization, predominantly on internal and external hip rotation, and the patient was unable to walk. 

**Figure 1:**
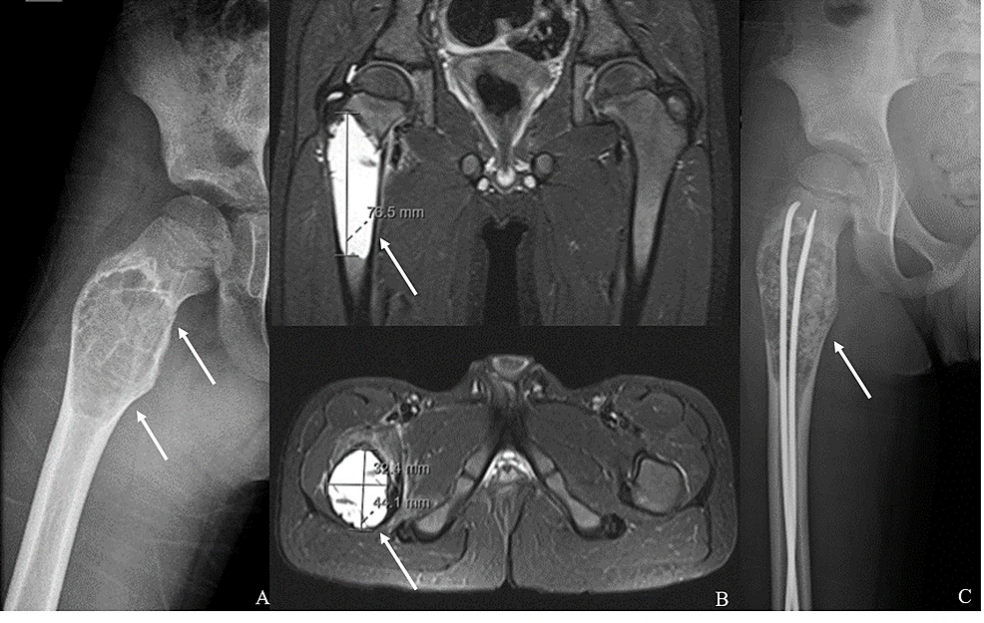
A) Radiograph: cystic lesion of the proximal right femur; B) MRI: expansive lesion, on longitudinal and transversal planes; C) Radiograph after surgery: intramedullary nails image

Radiographs revealed a cystic lesion on the proximal right femur, with thin sclerotic margins (“eggshell”), nearby the great trochanter, and two longitudinal fractures in its anterior and superior strand (Figure 1A). A CT highlighted the size and characteristics of the lesion: cystic lesion with an internal bone septum, sizing 77 x 44 x 32 mm, with fluid nearby the lines of fracture and right hip intra-articular effusion. Given the probable diagnosis of an aneurysmal bone cyst, the patient was admitted to the orthopedic department. Pain control and further imagiological characterization were performed: MRI findings supported the suspected diagnosis and this exam was used for guidance on surgical planning (Figure 1B). The patient was submitted to surgical resection by bone curettage, followed by cryotherapy, bone grafting, and intramedullary nailing (Figure 1C). Histological analysis revealed characteristics compatible with an aneurysmal bone cyst without malignant cells.

PMR evaluation was performed on the sixth postoperative day. The pain was graduated by the Numeric Scale of Pain (NSP) which ranges from 0 to 10 [13]; ROM was evaluated by goniometry [14], and muscular strength was evaluated by manual muscle testing and graded according to Medical Research Council scale [15]. Functionally was subjectively accessed. By that time, the patient reported pain on the right hip (7/10 on NSP); physical examination revealed an impairment on active right knee ROM (-25º to 80º) and a muscular strength decrease on right knee flexion (3/5), extension (2/5) and on right plantar flexion and dorsiflexion (4/5). Other joints of both inferior limbs had complete ROM, except for the right hip which evaluation was limited by patient cooperation. Also, muscular strength was graded as 5/5 on all left inferior limb segments. The patient was able to walk with a walker and minimal load on the RIL, having some functional dependence.

After discharge on the following day, a daily PMR outpatient program was started. The goals of the rehabilitation program were pain control, scar desensitization, hip and knee ROM increase, RIL muscular strengthening, balance and gait training, and autonomy and participation recovery. The sessions included a combination of manual therapy, play-based neuromuscular reeducation, proprioceptive and gait training. Manual therapy was performed for scar mobilization and desensitization, ROM recovery, and isometric strength training of the quadriceps, external hip rotators, abductors and extensors, progressing to isotonic muscle strengthening. Gait training encompassed progressive load on the affected limb: partial weight-bearing and subsequent full weight-bearing after three weeks and six weeks, respectively. The patient was monthly evaluated and the PMR program was adjusted according to the clinical evolution, lasting for four months. A significant improvement in pain, scar characteristics, hip and knee ROM, muscular strength, and gait ability was found (Table 1). On the clinical follow-up six months after the programs’ conclusion, the patient had no musculoskeletal symptoms, sequelae or limitations and returned to his previous lifestyle and daily-life activities (both at home and in school). The patient is still undergoing regular clinical follow-up, scheduled for at least two years past the surgery.

**Table 1:** Patient clinical evolution aROM: active range of motion; cm: centimeters; DF: dorsiflexion; E: extension; F: flexion; IPP: inferior patella pole; LLP: lower limb perimetry; MRCs: Medical Research Council scale; MS: muscle strength; NE: not evaluated; NSP: Numeric Scale of Pain; PF: plantar flexion; SPP: superior patella pole.

Evaluated parameters	Day before discharge (30/10/19)	First appointment (02/12/19)	Second appointment (02/01/20)	Third appointment (27/01/20)	Fourth appointment (24/02/20)	Fifth appointment (08/09/20)
Pain (NSP)	7/10	0/10	0/10	0/10	0/10	0/10
Scars	NE	Slightly adherence, with peri cicatricial hypoesthesia	Not adherent; not painful	Not adherent; not painful	Not adherent; not painful	Not adherent; not painful
LLP 15 cm above SPP – right	NE	NE	30.5 cm	31.2 cm	31.5 cm	34.0 cm
LLP 15 cm above SPP – left	NE	NE	32.0 cm	32.0 cm	32.0 cm	35.0 cm
LLP 10 cm above SPP – right	NE	26.5 cm	27.1 cm	28.0 cm	28.0 cm	29.5 cm
LLP 10 cm above SPP – left	NE	28.0 cm	28.1 cm	28.5 cm	28.5 cm	30.0 cm
LLP 10 cm below IPP – right	NE	22.1 cm	NE	22.5 cm	22.7 cm	24.0 cm
LLP 10 cm below IPP – right	NE	22.5 cm	NE	23.0 cm	23.0 cm	24.5 cm
aROM right hip flexion	NE	125º	125º	125º	125º	125º
aROM right hip extension	NE	30º	30º	40º	40º	40º
aROM right hip external rotation	NE	45º	45º	45º	55º	55º
aROM right knee flexion	80º	120º	120º	120º	130º	130º
aROM right knee extension	-25º	-10º	-10º	-5º	0º	0º
MS right hip flexion (MRCs)	NE	4/5	4/5	4/5	5/5	5/5
MS right knee flexion (MRCs)	3/5	4/5	4/5	4/5	5/5	5/5
MS right knee extension (MRCs)	2/5	2/5	2/5	2/5	5/5	5/5
MS right ankle DF (MRCs)	4/5	5/5	5/5	5/5	5/5	5/5
MS right ankle PF (MRCs)	4/5	5/5	5/5	5/5	5/5	5/5
Gait ability	Possible, with walker	Possible, with two crutches	Possible, with one crutch	Autonomous	Autonomous	Autonomous

## Discussion

We report a pediatric case of an aneurysmal bone cyst that presented with sudden onset of pain due to a pathological fracture. Despite infrequent, PMR doctors should be aware of this entity, especially due to its challenging clinical presentation, differential diagnosis, and specific therapeutic needs.

Regarding the differential diagnosis of aneurysmal bone cysts, namely malignant bone tumors, both imagiological and histological findings are important to rule out a variety of lesions, specifically fibrous dysplasia, eosinophilic granuloma, giant cell tumor, non-ossifying fibroma, osteoblastoma, myeloma, chondroblastoma, and telangiectatic osteosarcoma [5,7]. In this case, both imagiological characteristics and histological reports confirmed a primary aneurysmal bone cyst, with the absence of malignant cells on histological examination.

Concerning the surgical treatment approach, the patient was submitted to bone curettage of the lesion followed by bone grafting, which is the standard of care [5]. Also, cryotherapy was performed, as an adjuvant, in order to reduce the risk of recurrence. Intramedullary nailing was an important part of the surgical procedure to fix pathological fractures. Post-surgery impairment, both in relation to the primary lesion and to surgical trauma, was addressed with a tailored PMR program. Indeed, PMR programs are of extreme importance and thus, they can contribute to symptom resolution and the ability to return to daily-life activities. Despite the negligible literature regarding what type of PMR program is most adequate, the hitherto evidence highlights its importance in morbidity reduction [12].

## Conclusions

Aneurysmal bone cysts are a rare entity that may lead to significant clinical and functional impairments. We reported a pediatric case of this entity that had a sudden presentation with pain due to a pathological fracture, in which surgical and rehabilitation treatment were performed with full recovery. PMR program was of utmost importance, both for clinical and functional improvements, so we provide an insight into our tailored treatment plan and its outcomes. Indeed, this is a paradigmatic case in which a rare condition with a rare presentation was displayed after several others were ruled out, requiring a multidisciplinary approach. 
